# Mite non‐reproduction is not a consequence of the brood removal behavior of varroa sensitive hygiene honey bee colonies (*Apis mellifera*)

**DOI:** 10.1002/ece3.11595

**Published:** 2024-06-25

**Authors:** Lina Sprau, Kirsten Traynor, Birgit Gessler, Martin Hasselmann, Peter Rosenkranz

**Affiliations:** ^1^ University of Hohenheim Stuttgart Germany; ^2^ Department of Livestock Population Genomics, Institute of Animal Science University of Hohenheim Stuttgart Germany

**Keywords:** *Apis mellifera*, mite reproduction, *Varroa destructor*, varroa resistance, varroa sensitive hygiene

## Abstract

A sustainable solution to the global threat of the *Varroa destructor* mite is the selection of varroa‐resistant honey bee (*Apis mellifera*) colonies. Both “mite non‐reproduction” (MNR) and “varroa sensitive hygiene” (VSH) appear to be promising selection traits for achieving the goal of a resistant honey bee. MNR describes colonies that have a high number of non‐reproductive mites (no offspring, no males, or delayed development of mite offspring). High numbers of non‐reproductive mites have been observed in selected colonies, but the mechanism behind this trait has not yet been identified. The specialized hygienic behavior of selected honey bees, called VSH, is the removal of varroa‐infested brood. These traits were thought to be linked by VSH bees preferentially removing reproductive varroa females leaving only non‐reproductive mites behind in cells and thus creating colonies with high levels of MNR. To further investigate this link, we used an experimental setup and data sets from a four‐year selection project designed to breed for MNR and VSH colonies. In addition, we sought to answer the question of whether non‐reproductive mites are a direct consequence of worker removal behavior. To test this, we artificially induced removal behavior, and after providing the mite with enough time to re‐enter another cell, we opened all capped cells, relocated the mites, and evaluated their reproduction. As shown in previous studies and in this study, VSH had no effect on MNR levels. Also, the induced removal behavior did not lead to non‐reproduction in the subsequent reproductive cycle post interruption. We thus concluded that breeding for non‐reproductive mites does not automatically breed for VSH behavior and worker removal behavior does not cause subsequent reproductive failure of the mites forced to flee and find a new cell for reproduction.

## Graphical abstract

The specialized hygienic behavior of selected honey bees, called varroa sensitive hygiene (VSH), was thought to cause high levels of non‐reproductive mites. To further investigate this link, we used the experimental setup and data sets from a four‐year selection project designed to breed mite non‐reproduction (MNR) and VSH colonies. However, our results show that breeding for non‐reproductive mites does not automatically breed for VSH behavior and removal behavior is not linked to the reproductive failure of the mites.

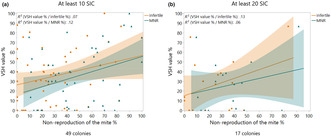

## 1 INTRODUCTION

The varroa mite *Varroa destructor* is one of the leading causes of worldwide colony losses of the western honey bee *Apis mellifera* (Neumann & Carreck, 2010; Rosenkranz et al., 2010; Traynor et al., 2020). To ensure the survival of their colonies, beekeepers have to treat several times during the year to reduce varroa infestations (Hernandez et al., 2022; Jack & Ellis, 2021). The combination of the parasite feeding on adults during the dispersal phase and on the brood during its reproductive phase, in addition to its transmission of multiple bee viruses, results in high losses of bee colonies without proper varroa management (Bowen‐Walker et al., 1999; Martin, 2001). A sustainable approach that would reduce the need for intervention via medication (varroacides) is selection for varroa tolerance or resistance in honey bee colonies (Dietemann et al., 2012). Many research and breeding programs are selecting characteristics that enable colonies to cope naturally with the varroa mite. Different approaches and selection criteria have been identified. One approach is natural selection where survival stock without treatment is used for breeding purposes (Blacquière et al., 2019; Fries et al., 2006; Locke, 2016b; Luis et al., 2022). This tactic has advantages such as lower work effort in selection, as it only requires minimal colony evaluations and a large enough starting population to compensate for the high colony losses. However, much of the stock produced via such methods has characteristic traits incompatible with commercial beekeeping such as high rates of swarming and small colonies (Guichard et al., 2023; Le Conte et al., 2007; Loftus et al., 2016). These surviving stocks seem to reach an equilibrium with their environment, both with the varroa population of the area and the viruses transmitted. When relocated to other regions, the varroa tolerance often fails to provide sufficient resistance and so the untreated colonies collapse (Büchler et al., 2014; Correa Marques et al., 2002). A different approach is breeding for specific traits linked to reduced varroa populations in colonies (Büchler et al., 2010, 2022; Kovačić et al., 2020; Mondet et al., 2020; Rinderer et al., 2010). With meticulous work and ongoing evaluation, resistance through breeding has been achieved in small‐scale operations, as well as in research‐focused apiaries such as those in Baton Rouge (Le Conte et al., 2020). However, success on a larger scale where colonies no longer need varroa management has not yet been achieved.

### 1.1 Selection via breeding

Resistance breeding has mostly focused on four selection criteria: mite non‐reproduction or decreased mite reproduction (MNR or DMR; previously described as suppressed mite reproduction [SMR]) (Mondet et al., 2020; Von Virag et al., 2022), varroa sensitive hygiene (VSH) (Dietemann et al., 2013; Harbo & Harris, 2005), recapping (REC) (Oddie et al., 2018), and grooming (Pritchard, 2016; Russo et al., 2020).

A high proportion of non‐reproductive mites in a honey bee colony can lead to a flattening of the mite's population curve, as mothers fail to reproduce and lose their fecundity over time. This observation was first named SMR (Harbo & Harris, 1999; Harris & Harbo, 2000). In later studies, they realized they had a high removal rate of varroa‐infested brood cells and concluded that the non‐reproduction rates were thus a result of the specific removal of varroa‐infested brood cells. They thus advocated for a name change to varroa sensitive hygiene (Harbo & Harris, 2005). Because the triggers and mechanisms of VSH are still unclear, and it has yet to be shown that an actual suppression of reproduction through the bees or brood is occurring, a name change to mite non‐reproduction (MNR) was proposed (Mondet et al., 2020). This term includes all forms of non‐reproduction without implicating a cause (Eynard et al., 2020; Le Conte et al., 2020; Mondet et al., 2020). MNR describes colonies with a high proportion of non‐reproductive mites. However, the term MNR as used in Mondet et al. (2020) does not distinguish between infertile mites and mites that simply delay their reproduction, resulting in fewer adult female daughters being raised successfully; to encompass this state, the term infertility‐based and fecundity‐based decreased mite reproduction (DMR) was coined to cover this form of a less fecund mite with no reproduction at all and reduced mite reproduction (Von Virag et al., 2022). In this study, we focused on MNR as the term is defined in Von Virag et al. (2022) (synonym: infertility‐based DMR). MNR describes the scenario where no viable offspring emerge, including mites with no offspring (infertile), no males, only males, or delayed offspring development with no mature females (Table 1). However, we also decided to separately indicate the mites that did not lay any eggs at all (infertile). This means that only the mother mite was found in these brood cells. Highlighting the infertile mites is important because it can be assumed that infertility has a different physiological background compared to delayed or reduced reproduction. Consequently, such traits could be implemented quite differently in resistant honey bee populations (Locke et al., 2012). Even with delayed development, a daughter who completes the hardening of her exoskeleton could emerge with the bee as a virgin, then re‐enter a new cell, and mate with her son (Häußermann et al., 2020). This now mated mite can produce daughters and therefore contribute to the varroa population growth inside the colony. Due to a reduced number of mature daughter mites, MNR still reduces the mite's population growth, although potentially not to the same degree as infertile mites. Throughout this study we use the nomenclature as shown in Table 1.

**TABLE 1:** Definition of non‐reproductive mites as used in this study.

Abbreviation	Meaning
MNR (mite non reproduction)	Mites with presumably no viable daughter emerging. This includes mites with no egg laying (infertile), no male progeny, only male progeny, or delayed development of offspring
Infertile	Mite with no egg laying at all. The brood cell only contains the mother mite

### 1.2 Varroa sensitive hygiene

Honey bees exhibit a high level of social immunity (Cremer, 2019; Cremer et al., 2007), which refers to the collective defense system employed by social animals to protect the group from diseases or pathogens through behaviors and interactions that minimize infection risks. One form of this social immunity is highly specialized hygienic behavior to remove diseased brood (Spivak & Danka, 2021). Whereas general hygienic behavior was first selected to improve resistance to chalkbrood and American foulbrood, bees have also been selected specifically to remove varroa‐infested brood known as VSH (Mondet et al., 2020). The reproductive cycle of a varroa mite is thus interrupted by the removal of brood. While the pupae are removed by the workers, only a small percentage of mites are killed or damaged in the process (Bienefeld et al., 1999; Lodesani et al., 1996), with the remainder reinvading a cell and beginning a new bout of reproduction. As female mites have limited fecundity, and can only engage in a small number of reproductive cycles, VSH removal behavior can lead to lower rates of varroa population growth. Triggers of this removal behavior are still debated, though smell, damage‐dependent signals, signals given off by the mite and signals originating from the brood cells are believed to play a role (Ivanova & Bienefeld, 2023; Mondet et al., 2021; Nazzi et al., 2004; Schöning et al., 2012; Sprau et al., 2021, 2023; Wagoner et al., 2018).

### 1.3 VSH postulated to lead to MNR

After removal of the brood through VSH, the mite is most likely to enter a new cell. Kirrane et al. (2011) showed a failure of reproduction in the next reproductive cycle post interruption (Kirrane et al., 2011). Due to these findings, it was supposed that failure to reproduce was due to the brood removal behavior, linking VSH and MNR. In the Kirrane study, there were unusually high levels of non‐reproduction in all treatment categories including the controls. Thus, the results could have been due to cell manipulations and warrant further study. Despite differences in the potential mechanism of MNR (S and VSH, the terms are often used synonymously in some selection programs, leading to misunderstandings and methodological confusion.

The SETBie project (Selection and establishment of varroa‐resistant honey bees in Baden‐Württemberg, Germany) was a four‐year selection program focused on fixing VSH and the MNR traits in breeding stock. This project examined both traits in numerous colonies over a longer period of time, permitting a comparison of MNR (or infertile) with VSH data. Additionally, an experiment was conducted to examine the direct consequences of removal behavior on the reproductive ability of the varroa mite. Our experimental setup and datasets over multiple years allowed us to investigate two important linkages: (1) are the MNR (and infertile) values affected by the VSH values and (2) is the non‐reproduction of a mite a direct consequence of the colony's removal behavior. We find that these traits are not linked, nor is mite non‐reproduction a consequence of bees removing brood, because the mite forced to restart reproduction in a new cell shows the same reproduction pattern compared to undisturbed mites.

## 2 MATERIALS AND METHODS

The SETBie project was a varroa resistance project, where scientists worked together with local breeders to select for the varroa resistance traits VSH and MNR in Baden – Württemberg, Germany. As part of this project, data were collected from 2019 through 2022. The colonies were managed by their respective beekeepers in MiniPlus hive systems (Mini Plus Styropor®, 28 × 28 × 26 cm; six frames per hive body) during the year and were located all over Baden‐Württemberg. In this study, the MiniPlus management system was chosen for its widespread use among breeders. This system involves initiating the queen in a small unit and later transferring her to a standard colony. Since our evaluations were conducted shortly after the queen was established in the small colony, transferring her to a standard size colony was not possible. In order to incorporate diverse breeders and associations and establish a broad genetic foundation for this project, we employed various subspecies, including *A. mellifera mellifera* (14.2%), *A. mellifera carnica* (14.2%), *A. mellifera ligustica* (0.6%), and the popular hybrid *A. mellifera* Buckfast (71%).

The queens were artificially inseminated in the same year they were evaluated. Inseminations were done in May using selected stock with high levels of MNR and/or VSH. Some analyzed colonies (*N* = 398) were headed by single drone inseminated (SDI) queens, where the semen of only one drone was used for insemination. The other queens (*N* = 337) were multiple drone inseminated (MDI) with 8 μL of semen collected from multiple brother drones deriving from the same colony. 8 μL of semen is typically harvested from 8 to 12 drones, the variance is due to the quantity of sperm per drone. To avoid inbreeding, the drone and queen crosses came from different VSH lines. 43 colonies were open mated at mating stations with MNR selected stock (*N* = 43). Every year every beekeeper tried to rear around 10 sister queens from one mother queen. Altogether, 1402 colonies with selected queens were inseminated over the 4 years. We used 136 different mother queens that were inseminated with drones from 56 different donor colonies.

### 2.1 Evaluation of the MNR value

Every year, at the end of July, we evaluated the MNR of the colonies. To improve the number of cells infested with mites, our participating breeders infested each of their colonies with 180 mites collected from full‐size donor colonies 14–16 days prior to evaluation, which provides the mites with enough time to reproduce and re‐enter new brood cells. These mites were introduced into the hive for a higher infestation rate to better evaluate mite reproduction. The introduced mites were collected via the powder sugar shake method (Dietemann et al., 2013) and within a few hours placed on the top of the hive on a damp paper towel. Occasionally, some beekeepers cage the queen of highly infested colonies for a few weeks prior to harvesting mites. This practice creates a broodless colony, causing all mites to enter the dispersal phase and thus ensure a high level of mites for harvesting.

For the evaluation, 450 capped brood cells were inspected unless we found, 30 cells infested by a single mother mite (single infested cell = SIC), in which case we ceased sooner. Each cell was opened and the state of pupal development, amount of mother mites, and status of offspring were documented. We chose 450 cells as the outer limit for evaluation, a compromise between accuracy of the evaluation and limited impact on the colony, as opening additional cells in these small colonies could negatively impact colony growth. The MNR values are based on the RNSBB Protocol with minor alterations (Büchler et al., 2017). We evaluated reproductive failure using two measurements. The more commonly used value is the MNR value, which is a more generous definition that includes mites that fail to produce any offspring (infertile), no male offspring, only male offspring, or the oldest daughter is too young to reach maturity before the adult bee emerges. This means that in a cell containing pink‐eyed pupae (5–6 days after capping) a mite is counted as MNR if the oldest offspring in this cell is still an egg, in purple‐eyed pupae (7–9 days) if it is a protonymph and in black‐eyed pupae (10–12 days) if the oldest daughter is a deutonymph. The infertile value is a more conservative definition of non‐reproduction in the mite, where the mites fail to produce any offspring and do not lay any eggs. Thus, all infertile values are subsumed in the MNR value, but the more generous MNR has a broader characterization of mite non‐reproduction that includes the generation of non‐viable offspring.

Each year between 124 and 224 colonies were evaluated for MNR, so that during the four‐year project we analyzed 778 colonies. Out of the 778 colonies we could only use 285 in the MNR analysis because these colonies met the bare minimum of 10 SIC required to evaluate the reproductive capacity of the mite (Eynard et al., 2020). Only 1.9% (*n* = 15 colonies) had the desired mite count of 30 or more mites in single infested cells, making this an exceedingly rare occurrence.

### 2.2 Evaluation of the VSH value

After the MNR evaluation, we selected colonies to be evaluated for an additional VSH score. The VSH evaluation, where cells are directly infested with mites, is more time consuming and could thus only be conducted in a subset of colonies (*n* = 132 colonies). The VSH values were evaluated in August each year via the direct infestation method (Dietemann et al., 2013; Häußermann et al., 2020; Sprau et al., 2021).

The direct infestation method is done by introducing mites into freshly capped cells (capped no longer than 6 h). Freshly capped cells are chosen because the mite reproduces normally, and the developmental stage of the pupae can be clearly documented. Two treatment groups of 30 cells each were manipulated per hive: (i) individual mites were placed into the cell (mite) and (ii) cells were opened and closed without the introduction of a mite (control). The mites were collected via the sugar shake method from highly infested varroa donor colonies (Dietemann et al., 2013). After identifying freshly capped cells, a small incision was made, and the mite was placed close to the opening via a fine paintbrush. Attracted to the larvae, the mite walked into the cell and the cell cap was carefully closed again. Using this method, we can record the reaction of adult bees to the infested cell when the frame is returned to the colony. The control group, where no mites were placed into the cells, allows us to detect if the bees simply react to the cell disturbance rather than the mite. This direct infestation method requires skills to execute and so the control cells help demonstrate that the colony is truly recognizing mite‐infested cells rather than simply removing everything. All manipulated cells were on the same brood frame, though either side could be used, and the two treatment groups were randomly distributed on both sides.

### 2.3 Field test: Assessing the reproductive success of mites in the following reproductive cycle after they have been removed from sealed brood cells using artificially induced VSH behavior

This experiment was performed on five MiniPlus colonies located at the State Institute of Bee Research in Stuttgart, Baden‐Württemberg. Colonies had been treated to minimize varroa populations by removing all capped brood and applying an oxalic acid spray treatment to eliminate the remaining mites on adult bees at the start of August. Mites were collected via the powder sugar shake method from highly infested mite donor colonies without brood breaks. Mites were inserted into cells via the direct infestation method (see Section 2.2) and two groups of cells were created: (i) interrupted: 5 days post insertion of the mite into the cell, the cell cap and pupae were poked with a needle triggering hygienic behavior by workers. A duration of 5 days was chosen because under normal conditions this is when removal behavior is at its peak (Harris, 2007). All cells in the colony that were capped at the time of interruption were marked on a transparent sheet. The identification of already closed cells allowed us to exclude those cells for evaluation, as the mites need open cells for reinvasion. 8–10 days after poking the cells and triggering the hygienic behavior response, all cells that had been capped during this period were opened. Pupal developmental status, number of mites per cell, and the different status of mite offspring were documented. (ii) Undisturbed: Cells in the undisturbed group were also infested with mites by the direct infestation method and then the comb was placed back into the hive. The cells were analyzed 8 days later. The number of mites per cell and the status of mite offspring was documented. Afterwards the mites were removed from the cell and the comb was placed back into the hive.

In the interrupted cells only pink, purple, and black‐eyed pupae were used for the evaluation of non‐reproduction, because in earlier developmental stages the reproduction of the mite is not clearly visible. In the undisturbed brood, all were 8 days old, purple‐eyed pupae.

The number of undisturbed cells to be analyzed was certain, as the inserted number could be identified right away. The number of interrupted mites that could be relocated could not be predetermined, therefore we placed a higher number of mites inside of cells that were disturbed compared to undisturbed controls to ensure enough mites would be relocated. Mites could be removed by the bees, killed, or leave the colony in between cell opening via hygienic behavior and finding another cell to infest again.

Colony 5 was excluded from statistical analysis because a high re‐invasion of mites occurred during the time of the experiment; 101 more mites were found in the brood cells than inserted. This high number of mites is most likely due to a reinvasion of mites from the neighboring colony, which collapsed due to a high infestation of Varroa mites. The surviving mite‐infested workers most likely drift to a nearby colony, introducing unwanted mites. Under those circumstances, a clear distinction between introduced and reinvaded mites cannot be made, and thus this colony was excluded (Table A1).

### 2.4 Statistics

Statistical analysis was done with JMP®, Version 17.0.0. SAS Institute Inc., Cary, NC, 1989–2023. The removal mite rate is the number of empty brood cells cleared out by workers divided by the number of infested cells, without taking into account the removal of the control cells. The VSH value is the removal rate of the control subtracted from the removal rate of the mite‐infested cells, as it demonstrates that the bees only responded to varroa‐infested cells. If the removal rate of the control group exceeds that of the infested cells, the colony is assigned a value of zero since the removal rate cannot be negative. The infertile and MNR values show the ratio of non‐reproductive mites in a colony. The effect of VSH on infertile and MNR values was calculated with a bivariate linear regression. For the second experiment, a comparison of undisturbed and interrupted cells was done with a generalized linear mixed model (glmm) where the infertile and MNR values were binominal variables, undisturbed or interrupted were fixed effect and the colony was a random effect to make a cellwise comparison (Pirk et al., 2013). The model reads as follows: glmm (MNR ~ category + (1 | colony), family = binomial).

## 3 RESULTS

### 3.1 Effect of VSH values on the infertile and MNR values

**FIGURE 1:**
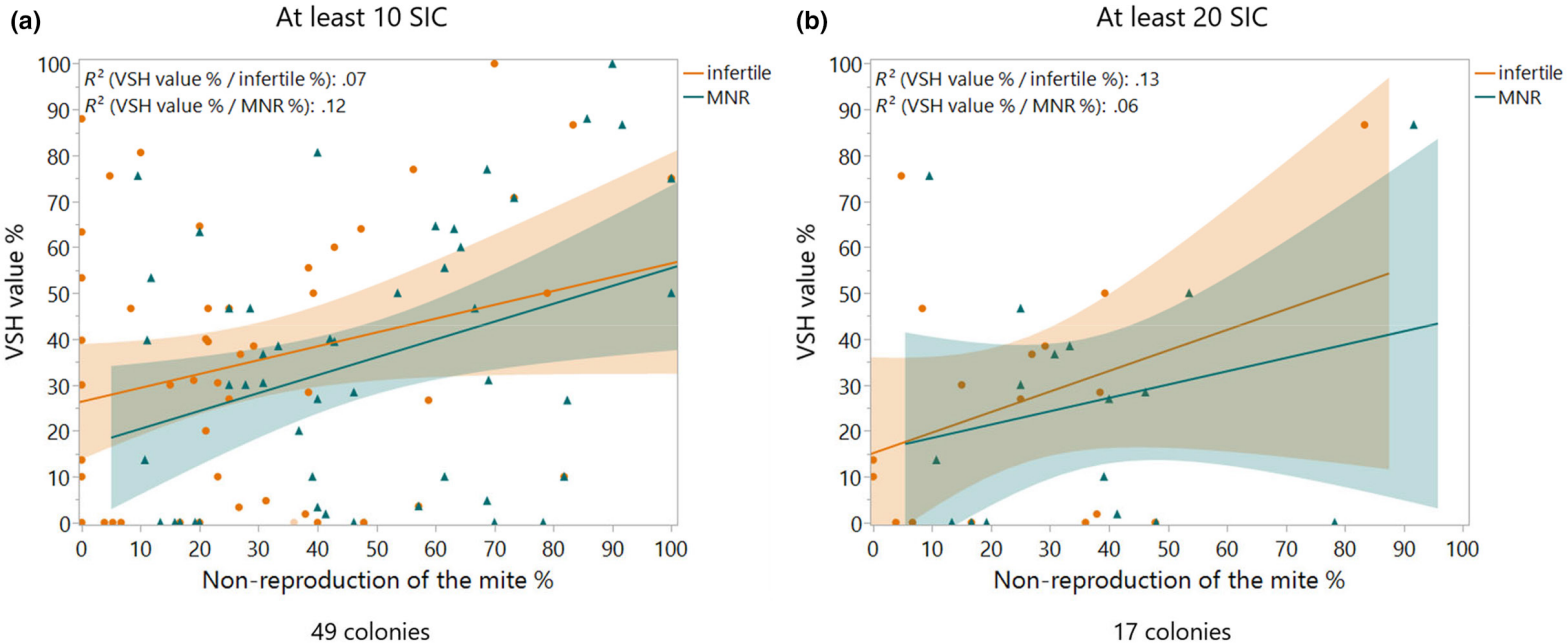
(a) Displayed are the VSH values (corrected for the removal rate of the control) in % compared to the infertile values (orange, circle) and MNR (green; triangle) for 49 colonies with 10 SIC. The linear regression of infertile: *y* = 26.36 + 0.3014 × *X* and MNR: *y* = 16.55 + 0.3891 × *X*. (b) Displayed are the VSH values (corrected for the removal rate of the control) in % compared to the infertile values (orange, circle) and MNR (green; triangle) for 17 colonies with 20 SIC.

It has been postulated that mite non‐reproduction or delayed reproduction is a result of mites being interrupted during their reproductive cycle from adult bees engaging in varroa‐sensitive hygiene. They then invade a new cell and fail to reproduce or delay ovipositing. We thus expected to see an effect of VSH behavior on the infertile and MNR values. However, they only weakly affected VSH values with an *r* ^2^ value between .07 (infertile) and .12 (MNR), and thus no clearly visible pattern indicating a strong link between these traits could be found (Figure 1). The VSH value significantly affects the MNR value (bivariate linear regression *p* = .0153; *F*_1,47_ = 6.33), but the *r* ^2^ value shows that it only explains up to 12% of the effect. With the infertile value, the link with VSH is even smaller (bivariate linear regression; *p* = .06; *r* ^2^: .07; *F*_1,47_ = 3.55). Only the colonies with at least 10 SIC can be used for the evaluation of infertile and MNR, thus of the 132 colonies evaluated for VSH, only 49 had a minimum of 10 cells infested by a single varroa mite, enabling us to also calculate infertile and MNR values. 18 colonies were only evaluated for VSH and therefore no MNR values were available. The different subspecies were represented with varying frequency in this data set of 49 colonies, 31 were *A. mellifera* Buckfast, 9 colonies *A. mellifera mellifera*, 8 colonies *A. mellifera carnica*, and 1 *A. mellifera ligustica* (Table 2). If a stricter threshold of at least 20 SIC are used, only 17 colonies can be evaluated. As seen with the analysis of 10 SIC, even at 20 SIC there is no visible link between infertile (bivariate linear regression *r* ^2^ = .13; *p* = .16; *F*_1,15_ = 2.19) or MNR (bivariate linear regression *r* ^2^ = .06; *p* = .34; *F*_1,15_ = 0.94) and VSH.

**TABLE 2:** Summary of colonies per evaluation.

Effect on VSH	Number of colonies	Years				Subspecies				Insemination		
		2019	2020	2021	2022	*Apis mellifera mellifera*	*Apis mellifera carnica*	*Apis mellifera ligustica*	*Apis mellifera* Buckfast	SDI	MDI	VSH mating station
10 SIC	49	7	14	13	15	9	8	1	31	25	24	0
20 SIC	17	0	5	5	7	5	3	1	8	8	9	
All colonies	114	12	33	32	37	21	20	4	69	53	59	2

Moreover, if we ignore the removal rate of control cells and just compare the removal rate of the varroa‐infested cells to the infertile and MNR values, we see similar results (infertile: bivariate linear regression; *p* = .02; *r* ^2^: .10; *F*_1,47_ = 5.5; *y* = 41.77 + 0.3927 × *X*; MNR: bivariate linear regression; *p* = .02; *r* ^2^: .10; *F*_1,47_ = 5.07; *y* = 35.32 + 0.3757 × *X*).

If VSH disrupts mite reproduction, then potentially we should find fewer mites in colonies with high VSH values and more mites in colonies with low VSH values. We thus examined all colonies, regardless of whether or not they met our SIC criteria of at least 10 infested cells (*n* = 114, Table 2). The number of SIC did not affect the VSH value or the mite removal rate (VSH value: bivariate linear regression; *p* = .06; *r* ^2^ = .03; *F*_1,112_ = 3.54; *y* = 44.78–0.6196 × *X*; mite removal rate: bivariate linear regression; *p* = .07; *r* ^2^ = .03; *F*_1,112_ = 3.16; *y* = 62.98–0.6496 × *X*). If we examine only the colonies with few SIC (<10; *n* = 65 colonies), we also see no connection with VSH value or mite removal rate (VSH value: bivariate linear regression; *p* = .7; *r* ^2^: .002; *F*_1,63_ = 0.11; *y* = 38.41 + 0.4278 × *X*; removal rate mite: bivariate linear regression; *p* = .93; *r* ^2^: .00; *F*_1,63_ = 0.01; *y* = 57.59 + 0.1171 × *X*).

While it would be expected that infertile and MNR values are highly interrelated, as colonies that have high infertile values automatically have high MNR values, the difference between the two values was up to 86% in the 49 colonies we additionally examined for VSH. When we consider all of the colonies evaluated during the SETBie project, we have colonies with zero infertile and 100% MNR. This difference between these two classifications methods for failed reproductive success in mites demonstrates that in some cases the non‐reproductivity is completely due to infertile mites and in other cases the lack of fertility is entirely due to missing males or delayed development of mite offspring.

### 3.2 Reproduction of mites after artificially induced VSH behavior

In this experiment we examined how brood removal behavior influences the non‐reproduction of the mite by stimulating adult bees to remove damaged brood, thus interrupting the mite's reproductive cycle. After 1 day, the status of brood removal was examined. All but two cells in total were cleared out within 24 h. We subsequently relocated the mites to investigate their reproductive success.

Between 35% and 71% of mites were found in brood cells with the pink, purple, and black‐eyed pupae states. Other studies suggest that mites that escape from hygienically removed brood cells immediately invade another cell if sufficient open brood cells are available (Boot et al., 1995; Table 3). Our colonies had sufficient open brood cells, as 8–10 days post inducing the hygienic behavior, we were able to inspect on average 408 ± 257 cells with pink‐eyed pupae, 344 ± 101 cells with purple‐eyed pupae, and 25 ± 29 cells with black‐eyed pupae. Thus, there were ample opportunities for potential cell reinvasion by the interrupted mites.

**TABLE 3:** The number of mites inserted and later found in the cells of pink, purple, and black‐eyed pupae in the different colonies and for the different treatment groups.

Colony ID	Treatment	Date cells opened	A	Cells opened				B	Mites in appropriately aged brood cells				Difference between inserted and found mites (A–B)^a^
			Number of mites inserted	Total	Pink pupae	Purple pupae	Black pupae	Total live mites found	Pink through black^b^	Pink pupae	Purple pupae	Black pupae	
Colony 1	Interrupted	25.08.22	95	1626	296	405	1	88	34	24	10	0	−7
	Undisturbed	19.08.22	50	27		27		24	24				−26
Colony 2	Interrupted	01.09.22	103	1689	849	230	28	124	76	39	33	4	+21
	Undisturbed	25.08.22	40	35		35		25	25				−15
Colony 3	Interrupted	02.09.22	54	1625	270	477	0	35	22	6	16	0	−19
	Undisturbed	26.08.22	50	34		34		24	24				−26
Colony 4	Interrupted	08.09.22	146	551	215	265	71	156	131	45	86	0	+10
	Undisturbed	02.09.22	35	27		27		20	20				−15

^a^ Difference is positive if we found more mites than we inserted. Although colonies had been treated, this experiment was conducted in August and September when rates of reinfestation are high.

^b^ Only single infested cells can be analyzed excluding in total 45 mites of the category interrupted and three mites from the undisturbed category from the analysis.

**FIGURE 2:**
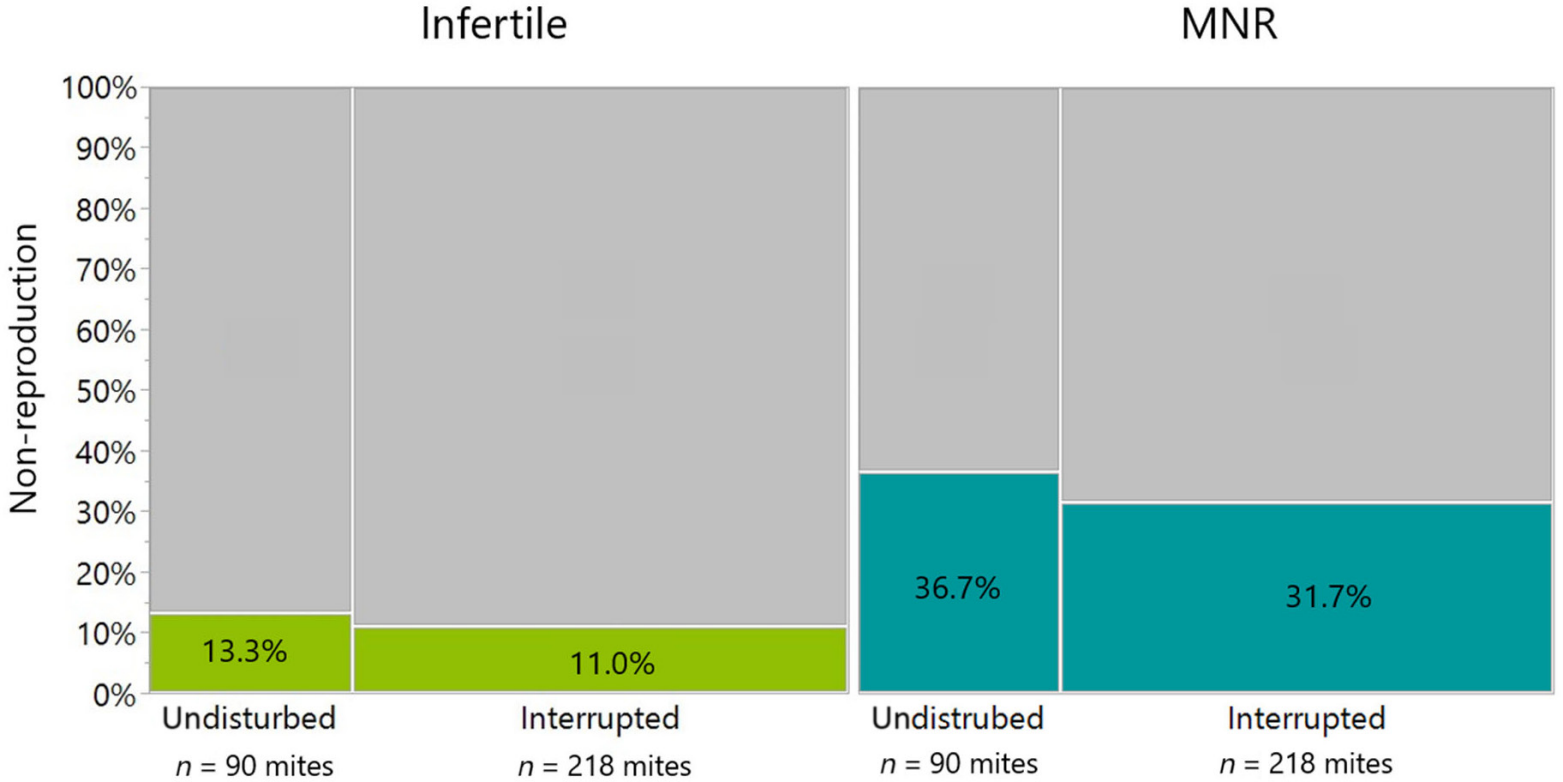
The number of mite non‐reproduction (infertile green and MNR blue) in undisturbed (*n* = 90 mites) and interrupted treatment groups (*n* = 218 mites) as a mosaic plot. There was no significant difference between undisturbed and interrupted mites in infertile or MNR (glmm, *p* > .05).

We found 12 of 90 (13.3%) infertile mites in the undisturbed treatment group, which is within the expected range of non‐reproductivity (Häußermann et al., 2020; Martin et al., 1997; Wendling et al., 2014). We found a similar rate of infertility among the 24 of 218 (11.0%) mites induced to invade another cell post hygienic brood removal, where adult bees were triggered to clean out the pupae pricked by a needle (glmm, *p* = .74, definition only infertile mites; Figure 2). When the broader MNR definition of non‐reproduction was used, we again found no significant differences (glmm, *p* = .80, Figure 2): 33 of 90 (36.7%) mites were infertile, missing male or had a delayed development of mite offspring in the undisturbed treatment group and 69 of 218 mites (31.7%) in the interrupted group.

**FIGURE 3:**
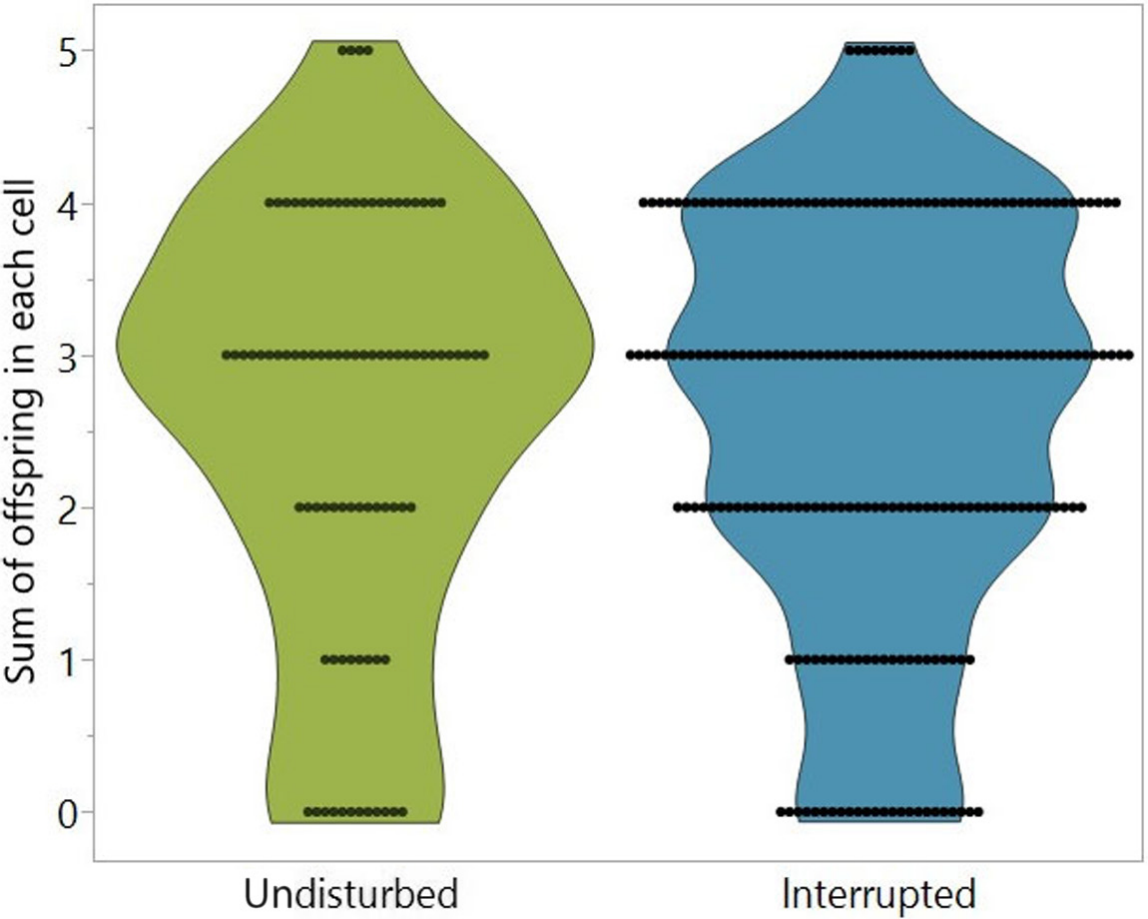
A violin plot of the sum of offspring per mother mite in undisturbed (blue) (*n* = 90 mother mites) and interrupted (green) (*n* = 218 mother mites) treatment groups, there was no significant difference between both groups (MM, *p* > .05).

There was also no significant difference (MM; *p* = .977; Figure 3) in the fecundity of the mites in the undisturbed group with 2.64 ± 1.38 offspring per mother mite (*n* = 90) compared to 2.64 ± 1.33 offspring per mother mite in the interrupted group (*n* = 218).

## 4 DISCUSSION

In the search for varroa resistance, many different traits linked to reduced mite viability have been examined. With regard to mite reproduction both, infertile and MNR (mite non‐reproduction; infertile, no male, delayed development of offspring) are useful indicators that can help predict a colony's ability to withstand varroa infestation (Buchegger et al., 2018; Mondet et al., 2020). The differences between these values are important to elucidate, and we believe both values are important to evaluate, as mite infertility (infertile) as well as mite immaturity (MNR) at the time of bee emergence lead to reproductive failure, reducing the parasite's population growth. However, MNR, while extremely useful for estimating the impact on mite population growth, is more vulnerable to examination errors, as the developmental status of the pupae and mite offspring have to be correctly identified.

It is often hypothesized that high levels of infertile or MNR mites are due to the interruptive opening of brood cells by VSH bees, which forces the mother mites to invade a new cell after already commencing reproduction. The supposed link between mite non reproduction via infertility or via delayed oogenesis and VSH (Harris et al., 2012; Le Conte et al., 2020) is challenged by both Eynard et al. (2020) and our current study. In our study, we used two different approaches to show that non‐reproduction in mites is not an effect of VSH behavior. We first demonstrated that there is almost no effect of the levels of VSH behavior on the infertile or MNR values. Secondly, we demonstrated that the failure of mites to reproduce is also not a direct consequence of the bee's removal behavior. Kirrane et al. (2011) suggested that the interruption of the reproduction cycle could lead to infertility of mites in the next brood cycle (Kirrane et al., 2011). Our results contradict this hypothesis, as we found equal levels of mite non‐reproduction in cells that did not experience hygienic behavior and mites forced to invade new brood cells after we induced hygienic behavior. A bias for the removal of solely reproductive mites, thus leaving behind only the non‐reproductive mites was also disproven in other studies, suggesting that these two traits are independent biological processes (Panziera et al., 2017; Sprau et al., 2021).

Some breeders and beekeepers who work on varroa‐resistant honey bees believe that if no mites can be found in a colony, it is automatically an indicator of enhanced removal behavior and thus high levels of VSH. Our results do not support this supposition, as the number of SIC (single infested cells) had no effect on VSH values, whereas if it was true, we would expect higher levels of VSH in colonies with fewer singly infested cells. While a low number of mites post colony infestation with 180 mites could potentially be a sign for VSH, it may be explained by other factors, such as mishandling of the introduced mites (i.e. high mortality, due to errors in handling), a low number of appropriately aged brood cells to invade, or high levels of grooming.

In this study, the MiniPlus management system was chosen for its widespread use among breeders. The MiniPlus system is commonly utilized in research to model colony dynamics due to its smaller size (around 4000 bees) and ease of handling. While it represents a complete hive with queen, drone, brood, etc., extrapolating results to standard hives is feasible but carries the risk of error and misinterpretation. We thus advocate that varroa resistance from proven lines is then evaluated in full size colonies.

The use of MiniPlus hives with their limited brood nests strongly restricts the number of available brood cells for evaluation. This forced us to limit our evaluations to opening 450 capped brood cells, as otherwise the colonies would have been too strongly impacted by this invasive method. There is an inherent limitation within our study. Breeders wanted to fix traits that improve varroa resistance. Selections of the mother and drone colonies for the following year often included colonies with high infertile or MNR values as well as high VSH values. Despite these selection choices, the link between infertile or MNR and VSH is low. The statistically significant but biologically almost irrelevant connection between infertile or MNR and VSH found in our study could be due to selecting for both traits when choosing the breeding stock rather than VSH having a direct effect on mite reproduction.

Various insemination techniques were employed in our study, with a focus on both single and multiple drone insemination methods. While single drone insemination provides a specific genetic contribution, colonies produced through this method often exhibit lower robustness. Well inseminated queens typically lead to more robust colonies (Cobey, 2007). To help ensure survival into the next season, approximately half of all queens received multiple drone insemination from colonies with the same drone lineage.

### 4.1 Limitations and potential improvements of current selection methods

Although we artificially infested our colonies with an extra 180 mites prior to evaluation, the rate of mite retrieval was surprisingly low. Of the 778 colonies evaluated, only 285 had 10 singly infested cells required as a bare minimum to evaluate infertile and MNR values. This suggests that the method requires optimization, as currently only a third of colonies analyzed provided the minimum of infested cells to correctly evaluate failures in mite reproduction. Less than 2% of all colonies had 30 or more mites. A potential improvement to the evaluation process could be delaying colony assessments until the following year. This approach allows for the evaluation of colonies at their full size, providing a more comprehensive understanding of their health and productivity. Additionally, it ensures that the mite population within the colonies is fully established, increasing the likelihood of finding a sufficient number of mites. However, implementing this change would prolong the evaluation timeline and may result in increased colony losses before assessment.

When searching for varroa resistance factors, both, MNR as well as the VSH method have their strengths. MNR can be implemented easily with just a brief training session by large groups of volunteers, allowing a large‐scale screening of many colonies using the normal brood development in the colony. However, MNR values have high variability in repeated evaluations of the same colony (Von Virag et al., 2022). Even though the heritability of the MNR traits seems promising (Gabel, Hoppe et al., 2023), the required workload is high. When using the MiniPlus system, very few colonies have high enough mite infestations to properly evaluate their reproduction. A large number of cells need to be opened and evaluated. When the evaluated colony still fails to make even the 10 SIC minimum, this can be frustrating for the beekeeper and researchers.

VSH, in contrast, records the direct reaction of the workers to varroa‐infested brood (Büchler et al., 2017; Dietemann et al., 2013). The direct infestation method is more precise, but even more time consuming than the MNR evaluation with a minimum of 2 h only to infest colonies and another hour to evaluate the VSH response after 8 days. It requires several training sessions prior to the experiment to learn how to correctly manipulate the colonies and infest mites into cells without injuring the freshly capped larvae. Due to this high work effort, we were only able to evaluate 132 colonies over the 4‐year research project. VSH values also fluctuate over the season, making it a difficult method for large‐scale breeding efforts (Tison et al., 2022).

Due to these inherent drawbacks in both methods, we highly recommend an optimization of the evaluation methods or switching to other, easier to apply selection criteria for resistance. Solidness of brood pattern or recapping could be easier to evaluate traits (Guichard et al., 2021). Evaluating the overall brood removal rate of cells over time and using artificial intelligence to analyze these changes automatically with software could potentially offer a simpler selection method. However, before implementing such tools in a large‐scale breeding operation, it is essential to demonstrate correlation of these traits with VSH and reduced mite infestation levels. Colonies in an apiary that maintain low mite populations while their neighboring colonies have high infestations could be another potential easy, though very imprecise, selection criteria.

The best solution for optimizing the evaluation of varroa resistance would be screening tests based on known molecular markers associated with desired traits, as it would be easier to screen large numbers of colonies. Currently these markers seem to vary across different varroa resistant populations. The genetic signatures underlying varroa resistance seem highly diverse. As the phenotype is difficult to evaluate, it makes links to genetic markers even more complicated and reliable markers are not yet known.

### 4.2 Infertile and MNR—Distinct traits?

Reproduction is a complex process. The triggers and mechanisms of infertility of mites or MNR are still unknown. We know that the activation of mite reproduction is triggered by signals from the freshly capped larvae (Frey et al., 2013; Rosenkranz & Garrido, 2004), thus a suppression signal produced by the larvae or pupae or lack of triggering signal most likely stops or delays the mite's egg laying process (Scaramella et al., 2023). These signals could be a chemical cue or the lack of an important nutritional prerequisite typically gained during feeding on the larvae or pupae. Mishandling of mites during artificial infestation of colonies to be assessed can also lead to non‐reproduction (Gabel, Scheiner, Büchler et al., 2023). It is probable that infertile and MNR values are driven by distinct mechanisms. Infertility is likely triggered by factors affecting the adult mites' ability to reproduce, while MNR may also result from issues such as offspring loss or developmental failure of the offspring, for instance, the removal of males during the recapping process (Gabel, Scheiner, Steffan‐Dewenter et al., 2023). These distinct mechanisms could also account for the variations observed in infertile and MNR values within the same colony.

### 4.3 VSH does not cause mite infertility or delayed development of offspring

Our results clearly show that the hygienic removal behavior does not lead to mite non‐reproduction, a consequence suggested in other studies (Kirrane et al., 2011). The results from our second experiment directly contradict the assumption of Kirrane et al. (2011) that interruption of reproduction via removal behavior results in a failure to reproduce in the next reproduction cycle. The Kirrane et al. (2011) study had a very high occurrence of infertility in all categories including the control group, suggesting that there may have been a methodological error, perhaps due to the mites being inserted up to 24 h after capping. Infertility in mites can be artificially induced by placing the mite into the cell long after capping (Frey et al., 2013; Garrido & Rosenkranz, 2003). The mite should not be inserted into the cell more than 6 hours after capping to guarantee normal reproductive behavior. In our study the removal behavior had no influence on the reproductive capability of the varroa mite. The interrupted as well as the undisturbed mites show similar rates of infertility of up to 13%, confirming rates of naturally occurring infertility in colonies (Häußermann et al., 2020; Martin, 1994; Martin et al., 1997). Interruption of reproduction via hygienic behavior had no impact on the number of offspring produced, thus VSH is not the cause of infertile mites or MNR.

The brood interruption undoubtedly disrupts the current cycle, and if consistently implemented, the Varroa mite population should not be able to grow. One limitation of our study is the small sample size, consisting of only 4 colonies. However, considering that we opened approximately 6500 cells (including the excluded colony 5), this was the maximum workload achievable within the constraints of this setting.

Results from both our experiments demonstrate that there is no strong link between VSH and infertile or MNR values. The non‐reproduction of the mite is not a direct consequence of removal behavior of the mite‐infested cells. Workers do not seem to differentiate between reproductive and non‐reproductive mites in infested cells, clearing them out at equal rates. Thus, VSH behavior does not lead to high infertile or MNR scores, which could occur if they only left non‐reproductive mites in the cells and removed the reproductive ones (Panziera et al., 2017; Sprau et al., 2021). Our results support the work of Eynard et al. (2020) and Büchler et al. (2022), who also found no correlation between VSH and infertile/MNR, when these traits are quantified successively within the same colony. Selecting for mite non‐reproduction does not automatically select for VSH or vice versa. While these traits are behavioral modifications in naturally resistant populations (Le Conte et al., 2020; Locke, 2016a, 2016b; Locke & Fries, 2011; Panziera et al., 2017), their usability in breeding efforts needs optimization, as currently the return for the massive time investment is too low to justify the cost.

## AUTHOR CONTRIBUTIONS

**Lina Sprau:** Conceptualization (equal); data curation (lead); formal analysis (lead); investigation (equal); methodology (equal); visualization (lead); writing – original draft (lead); writing – review and editing (equal). **Kirsten Traynor:** Validation (equal); writing – review and editing (equal). **Birgit Gessler:** Investigation (equal); methodology (equal); writing – original draft (supporting). **Martin Hasselmann:** Conceptualization (equal); validation (equal); writing – review and editing (equal). **Peter Rosenkranz:** Conceptualization (equal); supervision (equal); validation (equal); writing – review and editing (equal).

## FUNDING INFORMATION

The SETBie – project (Selection and Establishment of varroa‐resistant honey bees in Baden‐Württemberg) funds have been provided within the framework of the European Innovation Partnership “Agricultural productivity and sustainability” (EIP‐AGRI) as part of the action and development plan of Baden‐Württemberg 2014–2020 (MEPL III). Financing is provided by the state Baden‐Württemberg and the European Agricultural Fund for Rural Development (EAFRD).

## Supporting information


Appendix S1.

## Data Availability

The datasets generated during and/or analyzed during the current study are uploaded as Supporting Information.

## Appendix

**TABLE A1:** The number of mites inserted and later found in the cells of pink , purple , and black eyed pupae in the different colonies and for the different treatment groups in the colony 5.

Colony ID	Treatment	Date cells opened	A	Cells open				B	Mites in appropriately aged brood cells				Difference between inserted and found mites (A–B)^a^
			Number of mites inserted	Total	Pink pupae	Purple pupae	Black pupae	Total live mites found	Pink through black	Pink pupae	Purple pupae	Black pupae	
Colony 5	Interrupted	17.09.22	113	1021	39	385	54	214	83	13	63	7	+101
	Undisturbed	11.09.22	35	29		29		23	23				−12

*Note*: Colony 5 was excluded for the analysis due to a high invasion of mite by a collapsing neighboring colony.

^a^ Difference is positive if we found more mites than we inserted.
